# Vaccination with a *ZNF2*^oe^ Strain of Cryptococcus Provides Long-Lasting Protection against Cryptococcosis and Is Effective in Immunocompromised Hosts

**DOI:** 10.1128/iai.00198-23

**Published:** 2023-06-20

**Authors:** Tuyetnhu Pham, Yeqi Li, Wendy Watford, Xiaorong Lin

**Affiliations:** a Department of Plant Biology, University of Georgia, Athens, Georgia, USA; b Department of Microbiology, University of Georgia, Athens, Georgia, USA; c Department of Infectious Diseases, University of Georgia, Athens, Georgia, USA; Tulane School of Medicine

**Keywords:** asymptomatic infection, CD4 T cells, CD8 T cells, *Cryptococcus neoformans*, cryptococcal meningoencephalitis, immunization, morphogenesis

## Abstract

Systemic cryptococcosis is fatal without treatment. Even with the current antifungal therapies, this disease kills 180,000 of 225,000 infected people annually. Exposure to the causative environmental fungus Cryptococcus neoformans is universal. Either reactivation of a latent infection or an acute infection after high exposure to cryptococcal cells can result in cryptococcosis. Currently, there is no vaccine to prevent cryptococcosis. Previously, we discovered that Znf2, a transcription factor that directs Cryptococcus yeast-to-hypha transition, profoundly affects cryptococcal interaction with the host. Overexpression of *ZNF2* drives filamentous growth, attenuates cryptococcal virulence, and elicits protective host immune responses. Importantly, immunization with cryptococcal cells overexpressing *ZNF2*, in either live or heat-inactivated form, offers significant protection to the host from a subsequent challenge by the otherwise lethal clinical isolate H99. In this study, we found that the heat-inactivated *ZNF2*^oe^ vaccine offered long-lasting protection with no relapse upon challenge with the wild-type H99. Vaccination with heat-inactivated *ZNF2*^oe^ cells provides partial protection in hosts with preexisting asymptomatic cryptococcal infection. Importantly, once animals have been vaccinated with heat-inactivated or live short-lived *ZNF2*^oe^ cells, they are protected against cryptococcosis even when their CD4^+^ T cells are depleted at the time of fungal challenge. Remarkably, vaccination with live, short-lived *ZNF2*^oe^ cells in CD4-depleted hosts still provides strong protection to these hosts with preexisting immunodeficiency at the time of vaccination. This work raises hope for developing effective vaccines with long-lasting protection for individuals who are immunocompromised or could become immunocompromised later in life.

## INTRODUCTION

Cryptococcus neoformans is a ubiquitous environmental fungus found in soil, trees, and bird guanos (1, 2). This fungus enters the host through inhalation, and asymptomatic exposure to Cryptococcus is thus common in the general human population (3–5). The fungus either is cleared or stays dormant in the lungs. However, reactivation of dormant infections in individuals with impaired immunity due to AIDS or the immunosuppressive regimens used in transplant patients or for cancer therapy often leads to systemic cryptococcosis, which manifests as fatal cryptococcal meningoencephalitis (5–9). Even with the standard antifungal treatment, the mortality rates of cryptococcal meningoencephalitis range from 10% to 70% (10–18). Furthermore, relapse frequently follows treatment (19), pointing to the failure of eradication with the current antifungal therapies alone. Consequently, this disease claims hundreds of thousands of lives each year and is responsible for 15% of deaths in AIDS patients (13, 20). Developing vaccines that can prevent lethal infections or augment antifungal efficacy is thus important in combating this fatal disease.

To develop effective vaccines, it is necessary to identify immunogenic molecules or strains. It is known that morphogenesis profoundly shapes cryptococcal interaction with various hosts (21). This dimorphic fungus can grow in the yeast or the hypha/filament form (22). Yeast cells are immune elusive and highly virulent to a mammalian host (23), while filaments offer resistance to natural predators like soil amoebae (21, 24, 25), but they are rarely found in human or animal hosts. Strains that grow in the filamentous form under host conditions are attenuated in virulence in animal models of cryptococcosis (24, 26, 27). We previously discovered that the transcription factor Znf2 drives filamentation in C. neoformans (28, 29). Deletion of *ZNF2* abolishes hyphal growth and enhances virulence, while overexpression of *ZNF2* drives filamentation and drastically attenuates virulence (28, 30). More importantly, *ZNF2*^oe^ cells, either in a live form (at a dose of 1 × 10^6^ cells/animal) or in a heat-inactivated form (at a dose of 1 × 10^7^ cells/animal), elicit strong protective immune responses from the mammalian host (30). We hypothesize that the protective effect of strains overexpressing *ZNF2* is associated with increased antigen accumulation in the capsule of these cells (31). Those antigens are also present in wild-type cells, albeit at a much lower level (31). Consequently, even the heat-inactivated wild-type cells become immunoprotective with an increased vaccination dose (5 × 10^7^ cells/animal) (31). However, there are limitations in our previous studies, as the experiments were conducted in A/Jcr mice with normal CD4^+^ T cells, and the vaccinated animals were monitored for only 60 days after challenge due to practical reasons. Here, we aimed to determine (i) if protection by heat-inactivated *ZNF2*^oe^ cells can prevent relapse from occurring after challenge, (ii) if *ZNF2*^oe^ vaccination in hosts with preexisting asymptomatic cryptococcal colonization will have any adverse effects, and (iii) if vaccination with *ZNF2*^oe^ cells could provide protection to hosts when their CD4^+^ T cells are depleted (to mimic the immune status of AIDS patients).

## RESULTS

### Vaccination with heat-inactivated *ZNF2*^oe^ cells offers long-lasting protection with no relapse after H99 challenge.

**FIG 1:**
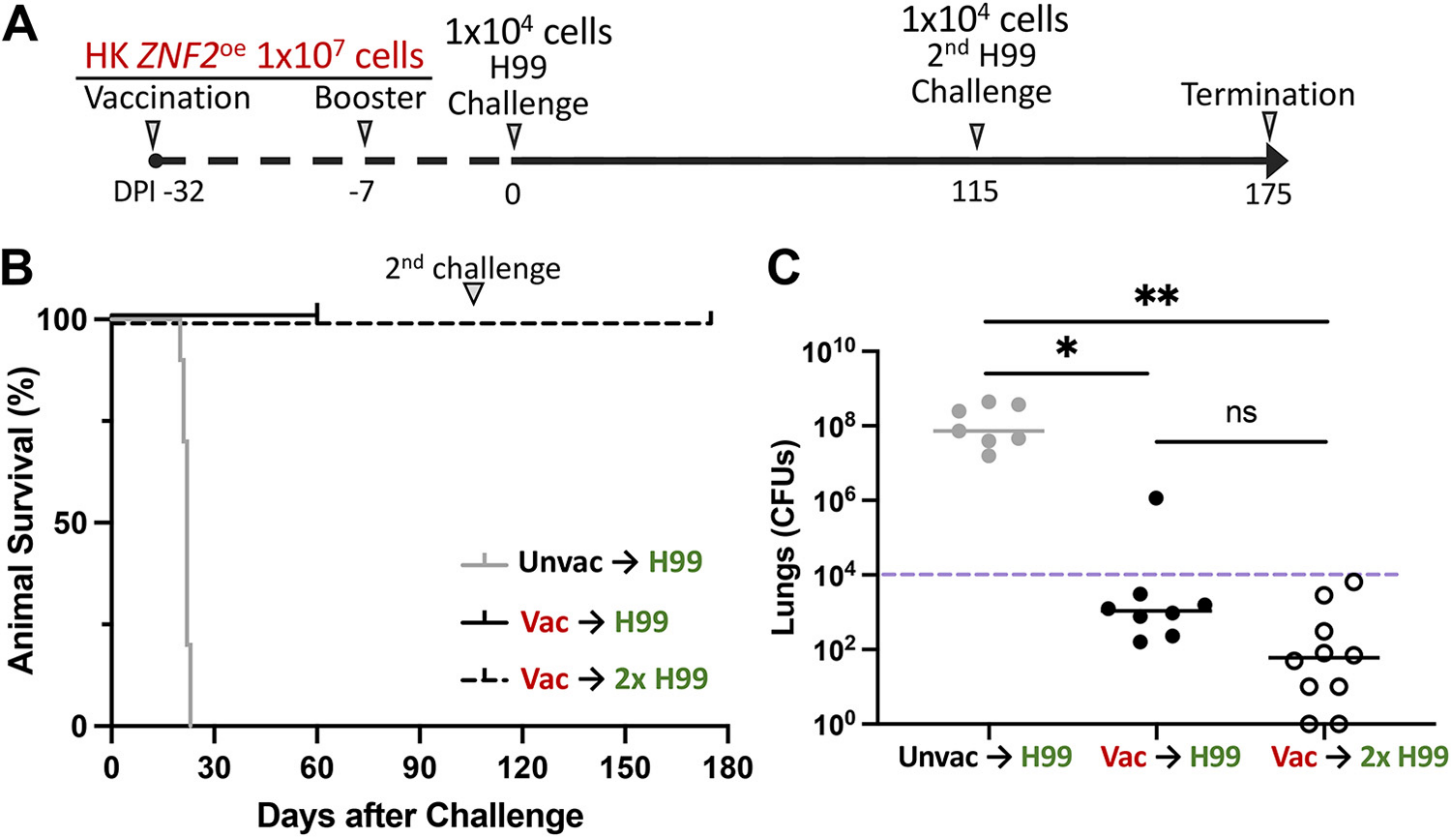
Vaccination with heat-killed *ZNF2*^oe^ cells protects mice from subsequent H99 challenge and relapse. (A) Vaccination regimen and H99 challenge. (B) Survival rates of 10 unvaccinated (Unvac) animals challenged with H99 and 10 HK *ZNF2*^oe^ cell-vaccinated (Vac) animals challenged by H99 once or twice. (C) Fungal burdens in the lungs of unvaccinated dying mice at the time of euthanasia and vaccinated mice at day 60 after the first H99 challenge (DPI 60) and at day 60 after the second H99 challenge (DPI 175).

Previously, we showed that mice vaccinated with heat-killed (HK) *ZNF2*^oe^ cells at a dose of 1 × 10^7^ cells/animal survived a subsequent challenge by live H99 when the experiment was terminated at day 60 postchallenge (30, 31). However, it was unclear if relapse could occur in these mice when the monitoring interval was extended. To address this question, we vaccinated mice with HK *ZNF2*^oe^ cells and then challenged them with live H99 cells as previously described (Fig. 1A). We monitored these mice for 115 days after the challenge. There were no signs of morbidity during the 115-day period (Fig. 1B). Since these mice have a median life span of less than 2 years (32), the result suggests that vaccination with HK *ZNF2*^oe^ cells offers these mice long-lasting protection after challenge and prevents relapse.

To determine if these vaccinated mice can become susceptible to cryptococcal infection again beyond 115 days after the first H99 challenge, we challenged these mice for a second time with H99. All mice survived the second infection when the experiment was terminated on day 175 (Fig. 1B). When the lung fungal burdens for the first challenge of H99 on day 60 (day 60 after the first challenge) and the second challenge of H99 on day 175 (day 60 after the second challenge) were compared, the lung fungal burdens in the group challenged twice were modestly lower than those in the group challenged only once, although the difference was not statistically significant (Fig. 1C). As expected, the unvaccinated mice had high fungal burdens in the lungs when they were euthanized at the clinical end points (31) (Fig. 1C). In the vaccinated group, however, animals survived. No fungal cells were recovered from the brains or kidneys of these vaccinated mice, indicating no extrapulmonary dissemination of H99. Thus, the results suggest that vaccination with HK *ZNF2*^oe^ cells can protect naive mice from two subsequent lethal Cryptococcus infections and prevent relapse over a prolonged observation period.

### The BC1076 cryptococcal strain causes persistent but asymptomatic pulmonary colonization.

Given that environmental exposure to Cryptococcus is universal, some individuals may have asymptomatic colonization. Here, we examined if vaccination of a host with asymptomatic colonization could cause any adverse effects.

**FIG 2:**
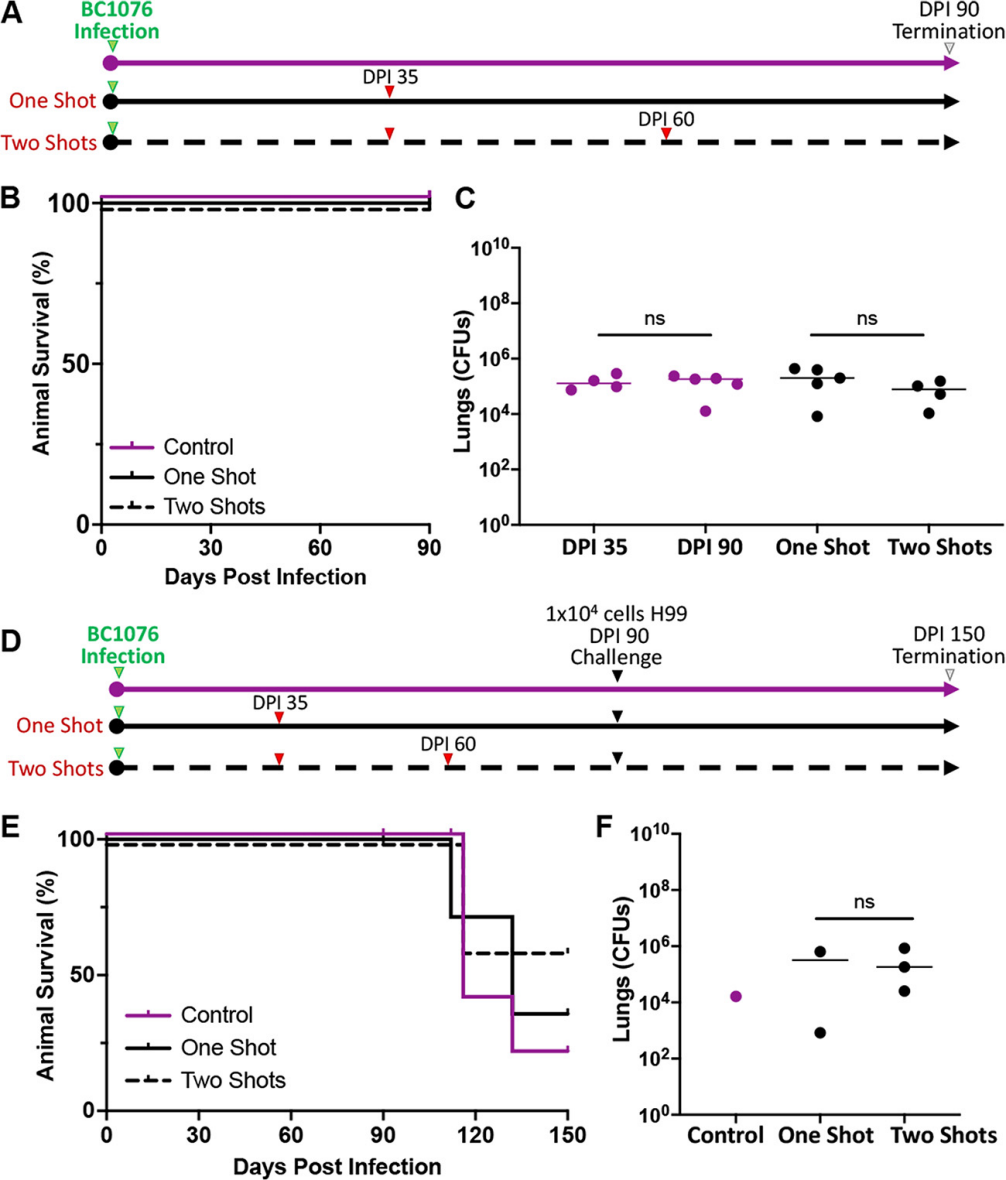
BC1076 causes persistent but asymptomatic pulmonary cryptococcosis. (A) BC1076 infection and vaccination regimens. (B) Survival rates of the three groups of mice (10 per group) from panel A. (C) Fungal burden of BC1076 in the lungs at DPI 35 and also at DPI 90 of three groups (unvaccinated, one shot, or two shots). (D) Regimens of BC1076 infection and vaccination and then challenge with H99. (E) Survival rates of the three groups of mice (10 per group) from panel D. (F) Lung fungal burdens of surviving mice in the three different groups at DPI 150.

Mice are generally hypersusceptible to virulent cryptococcal strains, and they rarely develop asymptomatic pulmonary colonization (33). The clinical isolate and reference strain H99, for example, causes fatal infection in various mouse strains even at a minuscule fungal inoculum. A previous study found that a serotype A cryptococcal strain, BC1076, does not cause mortality in mice in the inhalation infection model of cryptococcosis (34). The BC1076 fungal burden in the lungs increased from the original inoculum of ~10^4^ CFU/animal to ~10^5^ CFU/animal on day 35 postinoculation (DPI 35). Interestingly, the fungal load in the lungs was maintained at a similar level when the experiment was terminated at DPI 60 (34). Furthermore, this strain did not disseminate to the brain or the kidney, and the animals showed no sign of sickness at DPI 60 (34). To verify the suitability of this BC1076 strain for causing asymptomatic colonization in the lungs in a prolonged study, we inoculated mice with BC1076 at a dose of 1 × 10^4^ cells/animal intranasally and monitored these animals for 90 days postinoculation, during which time no mortality was observed (Fig. 2A and B, top magenta line). As expected, when examined at DPI 35, the median lung fungal burden was 1.29 × 10^5^ CFU (Fig. 2C), which is approximately 10-fold higher than the original inoculum, similar to what was previously reported. Fungal cells were not recovered from either the kidneys or the brains of any of the mice examined, indicating no extrapulmonary dissemination. At DPI 90, when the experiment was terminated, the median fungal burden in the lungs was 1.82 × 10^5^ CFU, similar to that at DPI 35 (Fig. 2C). Again, no fungal cells were recovered from either the kidneys or the brains of any of the examined mice. All mice looked healthy and showed no sign of illness on DPI 90. This result indicates that the BC1076 strain causes persistent but asymptomatic pulmonary cryptococcosis.

### Vaccination with heat-inactivated *ZNF2*^oe^ cells in mice with preexisting asymptomatic infection causes no adverse effect and provides some protection.

To examine the impact of the *ZNF2*^oe^ vaccine on mice with the preexisting but asymptomatic pulmonary colonization of C. neoformans caused by the BC1076 strain, we inoculated mice with BC1076 and randomly separated these mice into three groups (Fig. 2A): the control group (no vaccination), a group that received one dose of the HK *ZNF2*^oe^ cells at DPI 35 (one-shot group), and a group that received two doses of the HK *ZNF2*^oe^ vaccine, with one on DPI 35 and the other on DPI 60 (two-shot group). All three groups of mice survived to day 90 postinfection, when the experiment was terminated (Fig. 2B). On day 90 postinfection, all three groups of mice had comparable fungal burden in the lungs, with the median lung fungal burden of 1.82 × 10^5^ CFU for the control group, 2.0 × 10^5^ CFU for the one-shot group, and 0.78 × 10^5^ CFU for the two-shot group (Fig. 2C). Again, no fungal cells were recovered from either the kidneys or the brains from any of these groups. The result indicates that vaccination with HK *ZNF2*^oe^ cells (either one shot or two shots) does not adversely impact the preexisting asymptomatic colonization by BC1076.

To examine if vaccination with HK *ZNF2*^oe^ cells in mice with such persistent asymptomatic colonization by BC1076 is protective against a lethal cryptococcal infection, we challenged the three groups of mice with live H99 cells at 1 × 10^4^ cells/animal intranasally at DPI 90 and monitored them for an additional period of 60 days (to DPI 150) (Fig. 2D). In the inhalation infection model with naive mice, H99 first establishes lung infection and then disseminates to other organs, including the brain, and the infected mice become morbid around 3 to 4 weeks postinfection (31, 35). The control group with asymptomatic colonization without *ZNF2*^oe^ vaccination succumbed to H99 infection starting on DPI 116 (day 26 after challenge), similar to what we and others have observed previously in naive mice (31, 35) (Fig. 2E). Thus, the asymptomatic colonization by BC1076 did not provide any protection to the host from a subsequent lethal challenge with a virulent strain.

Two of the five mice with one dose of HK *ZNF2*^oe^ vaccination survived to the end of the experiment and showed no signs of morbidity at DPI 150. The median fungal burden in the lungs of these two surviving mice was 3.19 × 10^5^ cells (Fig. 2F). To determine the contribution of BC1076 cells to the total fungal burden, a sample of the homogenized tissues was plated on selective drug plates on which only BC1076 would grow. BC1076 cells were recovered from the lungs, representing 0.48% of the lung fungal burden. BC1076 cells were not recovered at all from either the brain or the kidney of any of these mice. Likewise, three of the five mice of the group with two HK *ZNF2*^oe^ vaccinations survived the H99 challenge and showed no signs of morbidity at DPI 150. The median fungal burden in the lungs of these mice was 1.82 × 10^5^ cells (Fig. 2F). Again, BC1076 cells were recovered from the lungs, representing less than 0.0001% of the lung fungal burden. BC1076 cells were not recovered at all from either the brain or the kidney of any of these mice. This result suggests that vaccination with HK *ZNF2*^oe^ cells does not cause any adverse effect in hosts with preexisting asymptomatic colonization, and it may provide modest dose-dependent protection from the H99 challenge.

### Hosts vaccinated with heat-inactivated *ZNF2*^oe^ cells were protected against H99 when their CD4^+^ T cells were depleted at the time of fungal challenge, and the degree of protection is dependent on the mouse strain.

Vaccination with live or heat-inactivated *ZNF2*^oe^ cells can protect mice from subsequent challenges by the highly virulent clinical strain H99 (30, 31). Vaccinating with HK *ZNF2*^oe^ cells twice at a dose of 1 × 10^7^ cells/animal or vaccinating with live *ZNF2*^oe^ cells once at a dose of 1 × 10^6^ cells/animal is highly protective in these models (30, 31). As most lethal cryptococcal infections occur in AIDS patients with insufficient CD4^+^ T cells, it is important to test the protective effect of *ZNF2*^oe^ vaccination in a CD4-deficient animal model.

**FIG 3:**
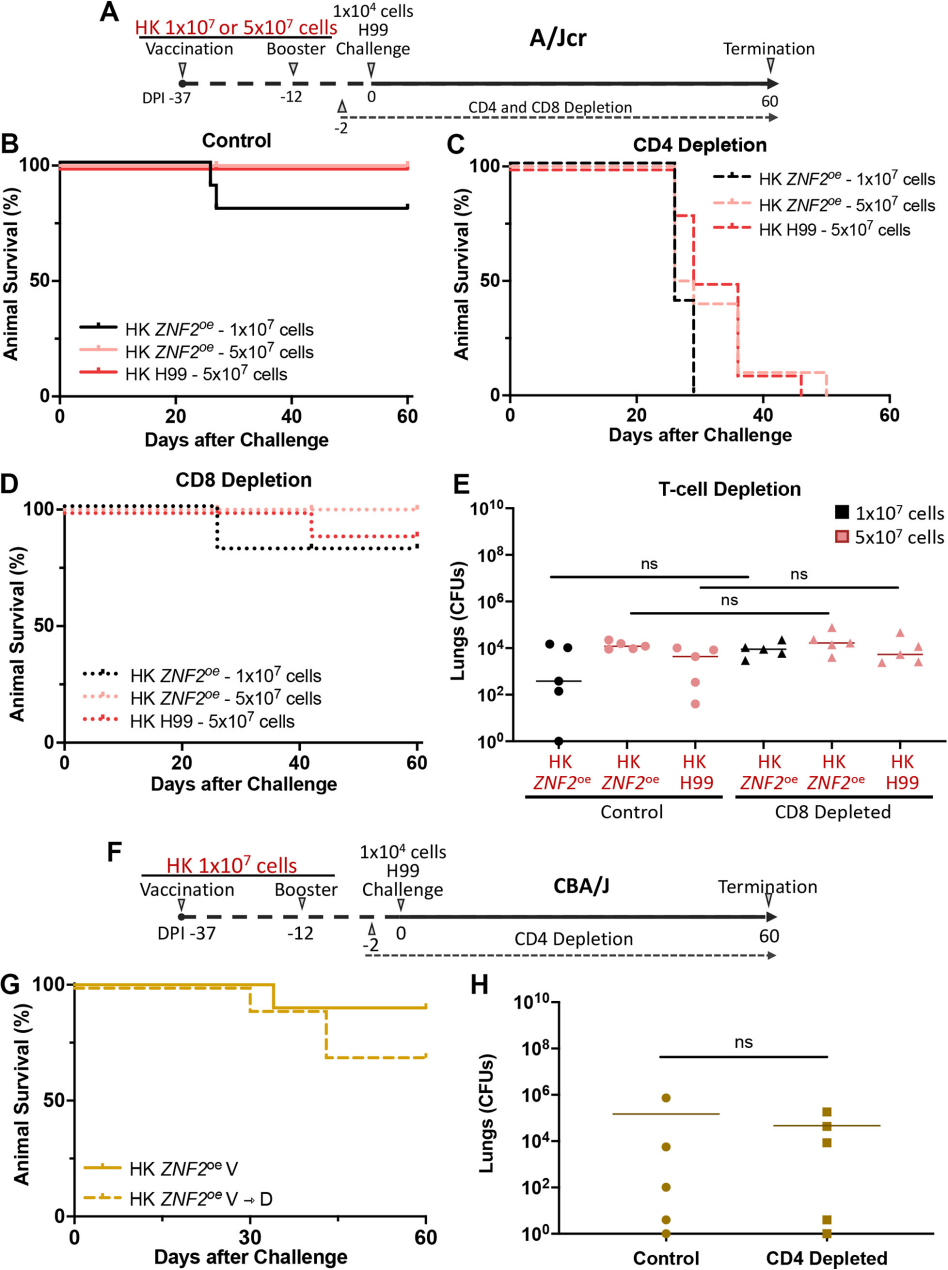
When CD4^+^ T cells are depleted, protection by heat-killed *ZNF2*^oe^ cells is host dependent. (A) Vaccination, challenge, and T cell depletion of A/Jcr mice. (B) Survival rates of the 10 control mice vaccinated with HK *ZNF2*^oe^ cells or HK H99 cells at the indicated doses and challenged with the wild-type strain H99. (C) Survival rates of the 10 CD4^+^ T-cell depleted mice vaccinated with HK *ZNF2*^oe^ cells or HK H99 cells at the indicated doses and challenged with the wild-type strain H99. (D) Survival rates of the 10 CD8^+^ T cell-depleted mice vaccinated with HK *ZNF2*^oe^ cells or HK H99 cells at the indicated doses and challenged with the wild-type strain H99. (E) Lung CFU in the surviving A/Jcr mice at DPI 60 after vaccination with HK *ZNF2*^oe^ cells or HK H99 cells at the indicated doses and challenge with the wild-type strain H99. (F) Vaccination, challenge, and T cell depletion in CBA/J mice. (G) Survival rates of 10 CBA/J mice vaccinated with heat-killed *ZNF2*^oe^ cells at the standard dose of 1 × 10^7^ cells/animal with or without CD4^+^ T cell depletion. (H) Survival rates of 10 CD4-deficient CBA/J mice vaccinated with heat-killed *ZNF2*^oe^ cells or live *sre1*Δ *ZNF2*^oe^ cells. V, vaccination; D, CD4^+^ T cell depletion.

Mice were vaccinated with HK *ZNF2*^oe^ cells twice at a dose of 1 × 10^7^ cells/animal as described above. Two days prior to the H99 challenge (DPI −2), mice were depleted of their CD4^+^ or CD8^+^ T cells using antibodies, and depletion was maintained for the study period. The degree of depletion of respective T-cell populations was monitored by flow cytometry (Fig. S1). These vaccinated and then T cell-depleted (V→D) mice were monitored for 60 days postchallenge, until the experiment was terminated (Fig. 3A). Depletion of CD8^+^ T cells had no impact on animal survival, as the survival rate for these mice is the same as that for the control group without any T cell depletion (Fig. 3B and D, black line). However, all animals with CD4^+^ T cell depletion reached clinical endpoints by DPI 29, with a median survival of 27 days (Fig. 3C, black line). This is comparable to the survival rate observed in unvaccinated naive mice infected with H99 (Fig. 1B). Thus, at this standard vaccination dose of 1 × 10^7^ cells/animal, HK *ZNF2*^oe^ cells failed to provide any protection to mice depleted of CD4^+^ T cells at the time of fungal challenge.

We showed previously that HK *ZNF2*^oe^ cells or HK H99 cells at the high dose of 5 × 10^7^ cells/animal provide strong protection to mice (31). Here, we tested to see if their protective effect at this high dose would remain in V→D mice depleted of CD4^+^ or CD8^+^ T cells. Again, depletion of CD8^+^ T cells did not have any significant impact on the protective effect of the HK *ZNF2*^oe^ cells or HK H99 cells at this high dose, in terms of either animal survival or fungal burden (Fig. 3B, D, and E, pink and red lines). However, V→D mice depleted of CD4^+^ T cells that received HK *ZNF2*^oe^ or HK H99 vaccines at the high dose still succumbed to the infection by DPI 50, with the median numbers of survival days being 27 and 32, respectively (Fig. 3C). Thus, vaccination with heat-inactivated cryptococcal cells even at this high dose provides only very modest protection to CD4-depleted mice.

So far, all the animal experiments had been performed using A/Jcr mice. Given that different mouse strains may react differently to vaccines and that A/Jcr mice are C5 deficient (36, 37), we decided to test the HK *ZNF2*^oe^ vaccine at the standard dose of 1 × 10^7^ cells/animal in a C5-sufficient CBA/J mouse model with or without CD4^+^ T cell depletion at the time of fungal challenge (V and V→D groups) (Fig. 3F). Depletion efficiency in CBA/J mice was confirmed by flow cytometry (see Fig. S1A in the supplemental material). CBA/J mice are known to be susceptible to H99 infection (38, 39). Surprisingly, once vaccinated with HK *ZNF2*^oe^ cells, most of the mice survived the lethal challenge by H99 even when their CD4^+^ T cells were depleted at the time of fungal challenge (Fig. 3G). When we examined the fungal burden of the surviving mice at DPI 60, the average lung fungal burdens for the V and V→D mice were 1.47 × 10^5^ and 4.67 × 10^4^, respectively (Fig. 3H), with a few mice completely cleared of this fungus. Furthermore, we did not recover any fungal cells from the brains or the kidneys of these mice. Thus, HK *ZNF2*^oe^ cells provide strong protection to CBA/J mice, even if they are depleted of CD4^+^ T cells at the time of an otherwise lethal cryptococcal challenge. Therefore, the protective effect of HK *ZNF2*^oe^ vaccination in mice depleted of CD4^+^ T cells depends upon the mouse strain background.

### Live mutated *ZNF2*^oe^ strains provide protection to A/Jcr mice when their CD4^+^ T cells are depleted at the time of fungal challenge.

Given that heat-inactivated cells at a high vaccination dose provided only weak protection to A/Jcr mice when their CD4^+^ cells were depleted at the time of fungal challenge, identifying a vaccination regimen that can work more effectively in this mouse strain is desirable. Live *ZNF2*^oe^ strains are more effective in providing host protection at lower doses than heat-inactivated *ZNF2*^oe^ cells (30, 31). However, the *ZNF2*^oe^ strain can replicate in the host in the first 2 weeks of infection, and it can persist in animals for weeks (30, 31). More than half of the mice infected with *ZNF2*^oe^ cells had not yet completely cleared the infection when examined on day 60 postinoculation (30). Thus, using a live *ZNF2*^oe^ strain as a vaccine could pose a potential risk to immunocompromised hosts.

**FIG 4:**
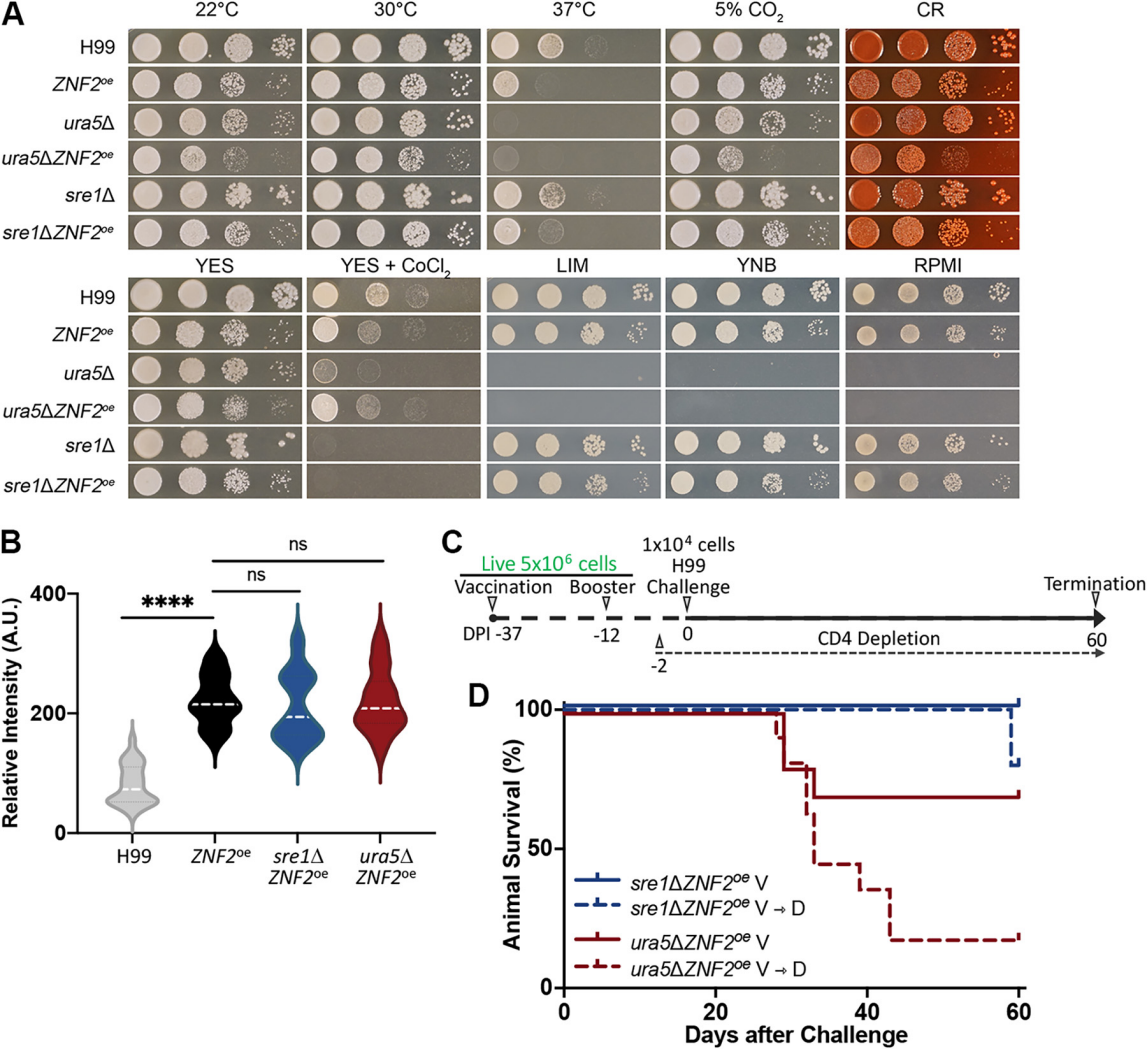
Live *ZNF2*^oe^ strains that cannot persist in the host are still protective to A/Jcr mice when their CD4^+^ T cells are depleted. (A) Growth of the wild-type H99 strain, the *ZNF2*^oe^ strain, the *ura5*Δ strain, the *ura5*Δ*ZNF2*^oe^ strain, the *sre1*Δ strain, and the *sre1*Δ*ZNF2*^oe^ strain under the indicated conditions: YPD at 22°C, YPD at 30°C, YPD at 37°C, YPD with 5% CO_2_ at 37°C, YPD with 0.05% Congo red at 30°C, YES at 30°C, YES with 0.5 mM CoCl_2_ at 30°C, YNB at 30°C, LIM at 30°C, and RPMI at 30°C. (B) Quantification of immunofluorescence intensity of cryptococcal cells from the wild-type strain H99, the *ZNF2*^oe^ strain, the *sre1*Δ*ZNF2*^oe^ strain, and the *ura5*Δ*ZNF2*^oe^ strain when probed with the protective serum. (C) Vaccination, challenge, and T cell depletion after vaccination with the live *ura5*Δ*ZNF2*^oe^ strain or *sre1*Δ*ZNF2*^oe^ strain. (D) Survival rates of 10 mice vaccinated with either *ura5*Δ*ZNF2*^oe^ or *sre1*Δ*ZNF2*^oe^ cells and challenged with wild-type H99 with or without CD4^+^ T cell depletion.

To minimize the risk posed by live *ZNF2*^oe^ cells, we generated *ZNF2*^oe^ strains with impaired ability to survive under conditions physiologically relevant to the host regardless of their immune status. To that end, we deleted the *SRE1* gene or the *URA5* gene from the *ZNF2*^oe^ strain background. *SRE1* encodes a transcription factor that regulates ergosterol biosynthesis under sterol-depleting conditions such as hypoxia (40). Sre1 is known to be required for cryptococcal meningoencephalitis (41). *URA5* encodes an orotate phosphoribosyltransferase, a critical enzyme involved in *de novo* biosynthesis of pyrimidines (42). The *ura5*Δ mutants are therefore auxotrophic for uracil/uridine and do not grow in minimal medium. As expected, the *ura5*Δ*ZNF2*^oe^ strain, like the *ura5*Δ strain, failed to grow on the minimal yeast nitrogen base (YNB) medium, low-iron medium (LIM), or the mammalian cell culture RPMI medium (Fig. 4A). Surprisingly, the *ura5*Δ*ZNF2*^oe^ and *ura5*Δ strains, even on the rich yeast peptone dextrose (YPD) medium, showed severe growth defect at 37°C, the human body temperature (Fig. 4A, top, center). Given that thermotolerance is a prerequisite for survival in the host, the *ura5*Δ*ZNF2*^oe^ strain is expected to live in the host only transiently. The *sre1*Δ*ZNF2*^oe^ strain and the *sre1*Δ strain, on the other hand, grew well under all these tested growth conditions except in the presence of cobalt chloride, a hypoxia-mimicking condition (Fig. 4A). Thus, we expect that both the *sre1*Δ*ZNF2*^oe^ strain and the *ura5*Δ*ZNF2*^oe^ strain will not be able to persist in the host regardless of their immune status, with the latter being able to survive only very transiently, given its auxotrophy and temperature sensitivity.

We previously showed that *ZNF2*^oe^ cells displayed increased antigens when probed with serum from protected mice vaccinated with HK *ZNF2*^oe^ cells or HK H99 cells (31). To examine if the *ura5*Δ*ZNF2*^oe^ strain and the *sre1*Δ*ZNF2*^oe^ mutants maintain the high abundance of antigens, we quantified the intensity of immunofluorescence using serum collected from protected mice vaccinated with HK *ZNF2*^oe^ cells. As expected, the fluorescence intensity derived from antigens present in *ZNF2*^oe^ cells that were recognized by the serum was much higher than that of the wild-type H99 cells, with a median value of 222.1 artificial units (AU) in *ZNF2*^oe^ cells, compared to 80.54 AU in H99 cells (Fig. 4B). The fluorescence intensity of the *ura5*Δ*ZNF2*^oe^ or the *sre1*Δ*ZNF2*^oe^ cells was comparable to that of the *ZNF2*^oe^ cells, with medians of 212.8 AU and 220.8 AU, respectively (Fig. 4B). These results suggest that deletion of the *URA5* gene or the *SRE1* gene from the *ZNF2*^oe^ strain does not affect the overall antigen abundance.

Next, we vaccinated A/Jcr mice with live *ura5*Δ*ZNF2*^oe^ and *sre1*Δ*ZNF2*^oe^ strains twice at a dose of 5 × 10^6^ cells/animal, as diagrammed in Fig. 4C. We then separated mice vaccinated with each strain into two subgroups, with one subgroup receiving antibodies to deplete their CD4^+^ T cells from day −2 before the challenge. All four groups of mice were then challenged with 1 × 10^4^ live H99 cells intranasally at DPI 0 (Fig. 4C). We found that 60% of animals vaccinated with the live *ura5*Δ*ZNF2*^oe^ strain survived the challenge with H99 by DPI 60. When CD4^+^ T cells were depleted, 20% of the mice survived (Fig. 4D). The median survival for this group with CD4^+^ T cell depletion was 33 days, which is still significantly better than survival of nonvaccinated mice (median survival was 26 days). In contrast, all animals vaccinated with the live *sre1*Δ*ZNF2*^oe^ strain survived the challenge by the end of the experiment at DPI 60. These animals appeared healthy and showed no symptoms of sickness at the time of termination. Remarkably, 80% of animals vaccinated with the live *sre1*Δ*ZNF2*^oe^ strain survived even with their CD4^+^ T cells depleted at the time of the H99 challenge (V→D group in Fig. 4D). When surviving mice were checked for the live *sre1*Δ*ZNF2*^oe^ strain, none was detected. This confirms that the live *sre1*Δ*ZNF2*^oe^ strain, unlike the *ZNF2*^oe^ strain, does not persist in the animals, and it shows promise as a vaccine for hosts who could become immunocompromised later.

### Vaccination with live *sre1*Δ*ZNF2*^oe^ strain provides greater protection to CBA/J mice than vaccination HK *ZNF2*^oe^ cells when hosts’ CD4^+^ T cells are depleted at the time of vaccination.

**FIG 5:**
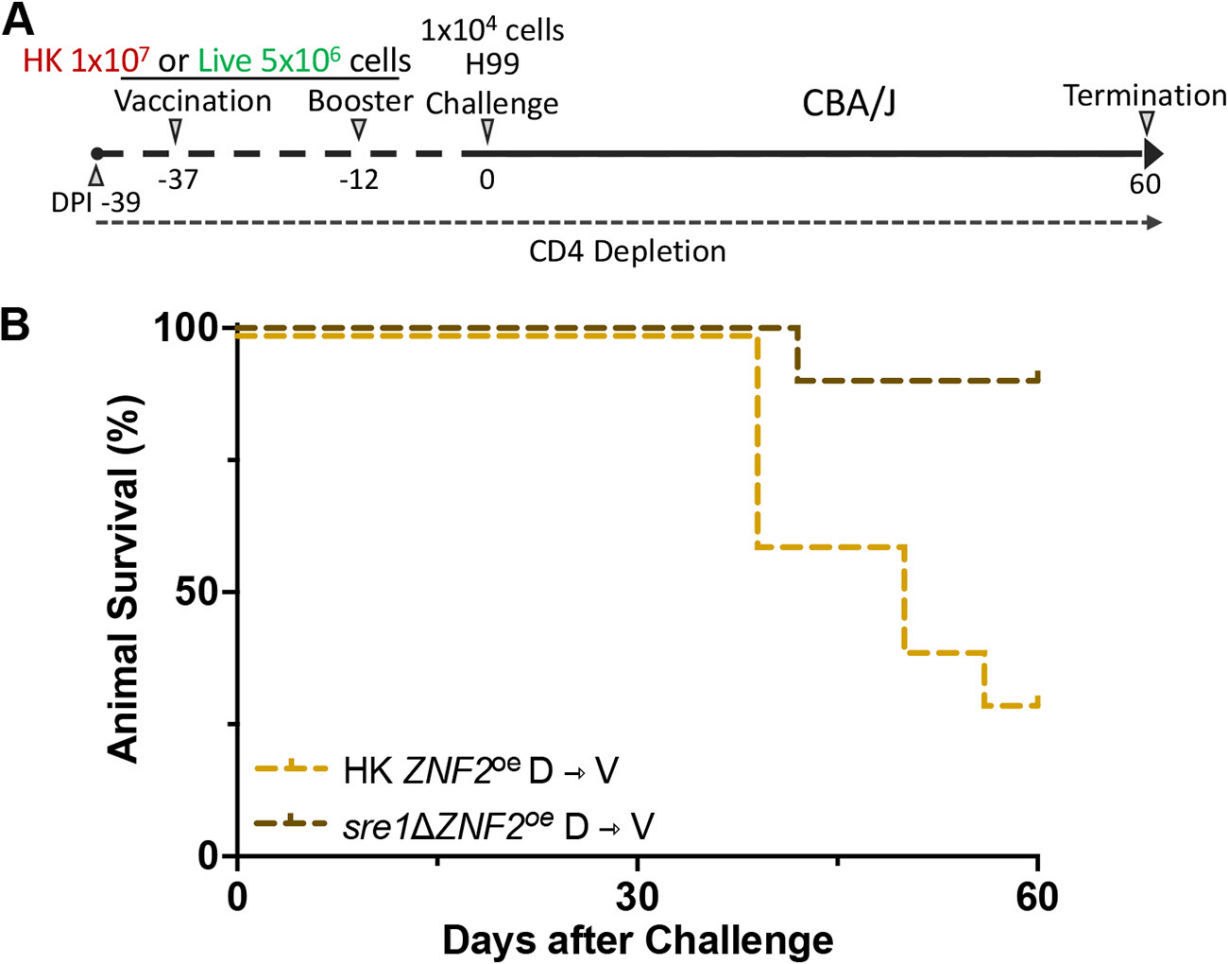
CD4-deficient CBA/J mice were protected from H99 when vaccinated with heat-killed *ZNF2*^oe^ cells or live *sre1*Δ*ZNF2*^oe^ cells. (A) Vaccination, challenge, and T cell depletion in CD4-deficient CBA/J mice. (B) Survival rates of 10 CD4-deficient CBA/J mice vaccinated with heat-killed *ZNF2*^oe^ cells or live *sre1*Δ*ZNF2*^oe^ cells. V, vaccination, D, CD4^+^ T cell depletion.

Because systemic cryptococcosis commonly occurs in AIDS patients with low CD4^+^ T cell count, we tested whether vaccinating hosts with an already low CD4^+^ T cell count with either the heat-killed or the short-lived *ZNF2*^oe^ strain will provide protection against otherwise lethal cryptococcosis. To this end, we first depleted CD4^+^ T cells in CBA/J and A/Jcr mice 2 days prior to vaccination (DPI −39) (Fig. 5A and Fig. S2). We then vaccinated and boosted these mice with HK *ZNF2*^oe^ cells at a dose of 1 × 10^7^ cells/animal or live *sre1*Δ*ZNF2*^oe^ cells at a dose of 5 × 10^6^ cells/animal. Next, we challenged the mice with wild-type H99 at 1 × 10^4^ cells/animal and monitored the infected mice for 60 days. CD4^+^ T cell depletion was maintained using anti-CD4 antibodies for the duration of the experiment as confirmed by flow cytometry (Fig. S1A). In the CD4^+^ T cell-depleted A/Jcr mice, vaccination with live *sre1*Δ*ZNF2*^oe^ cells significantly prolonged animal survival: the median number of days of survival in this group is 36, in contrast to the expected 22 medial survival days of unvaccinated mice without CD4^+^ T cell depletion (29–31, 43) (Fig. S2). Remarkably, 90% of CD4^+^ T cell-depleted CBA/J mice vaccinated with live *sre1*Δ*ZNF2*^oe^ cells survived the challenge (Fig. 5B). The result suggests that compared to the modest protection offered by vaccination with heat-killed *ZNF2*^oe^ cells, vaccination with short-lived *ZNF2*^oe^ cells is highly effective in hosts with a preexisting immune deficiency.

## DISCUSSION

Mouse models have been widely used to study cryptococcal infections. Mice are highly susceptible to infections by virulent cryptococcal strains, which is advantageous in the laboratory setting but also makes it challenging to study latent or asymptomatic infections. Despite their susceptibility to cryptococcal infections, mice of different backgrounds respond differently to vaccines (44–49). Here, we used both A/Jcr mice and CBA/J mice. A/Jcr mice are C5 deficient with an impaired innate immune system, and they are more prone to relapse. In contrast, CBA/J mice are C5 sufficient (50). Both mouse strains are widely used in Cryptococcus pathogenesis and vaccination studies (37, 44, 45).

In many other infectious diseases, virulence-attenuated strains are often used as vaccines (like the live attenuated TB vaccine). However, cryptococcal avirulent strains (e.g., stress-sensitive, temperature-sensitive, or acapsular mutants) are often not effective as vaccines. It is not clear if the lack of efficacy is a result of these strains’ being rapidly cleared by mice. If that is the case, one wonders if cryptococcal strains that can persist in animals but do not progress to systemic infections will be protective to the host against subsequent lethal challenges. Recently, Telzrow et al. reported a *mar1*Δ mutant that can cause chronic granulomatous infection in mice (33). Here, we found that the serotype A BC1076 strain caused persistent asymptomatic pulmonary cryptococcosis for the 90 days that we monitored. Remarkably, the fungal burden in the lungs remained constant during this entire study period. Thus, the BC1076 strain could provide another option for future investigation into cryptococcal latency and persistence, an underexplored but critical topic to understand cryptococcal colonization in the general population. Notably, the prior asymptomatic pulmonary colonization by the BC1076 strain does not provide host protection against future lethal infections, and it also diminishes the protective effect of later vaccination with HK *ZNF2*^oe^ cells. This is a factor that future cryptococcal vaccination research needs to consider and investigate further.

Given that the populations at high risk of systemic cryptococcosis are AIDS patients and individuals receiving solid organ transplants or immunosuppressive therapies, the ability to protect immunodeficient individuals is a must for any cryptococcal vaccines to be developed. Here, we tested the protective effect of *ZNF2*^oe^ vaccination in a CD4^+^ T cell-deficient animal model. We found that vaccination of A/Jcr mice with HK *ZNF2*^oe^ cells at the standard dose failed to provide any protection when these mice were depleted of CD4^+^ T cells prior to the lethal challenge, and only a weak protective effect was observed at the high vaccination dose in this model. In contrast, the majority of vaccinated CBA/J mice survived the lethal challenge by H99 even when their CD4^+^ T cells were depleted at the time of fungal challenge. These results suggest that once animals are vaccinated with HK *ZNF2*^oe^ cells, the hosts can be protected even though their CD4^+^ T cells are depleted after vaccination and the degree of protection depends on the mouse strain background.

As live strains are more effective in providing host protection than heat-inactivated cells, we then created *ura5*Δ*ZNF2*^oe^ and *sre1*Δ*ZNF2*^oe^ strains to be used in immunocompromised A/Jcr mice. Both *ura5*Δ*ZNF2*^oe^ and *sre1*Δ*ZNF2*^oe^ strains showed similar levels of host antigen recognition as the original *ZNF2*^oe^ strain based on immunofluorescence. The *ura5*Δ*ZNF2*^oe^ strain is auxotrophic and temperature sensitive, and this strain is not expected to survive or replicate in any mammalian host. The *sre1*Δ*ZNF2*^oe^ strain is defective in growth under hypoxia conditions, and the *sre1*Δ mutant is known to be incapable of causing cryptococcal meningitis (41). Vaccination with live *ura5*Δ*ZNF2*^oe^ cells provided strong protection in A/Jcr mice, but its protective effect in A/Jcr mice when their CD4^+^ T cells were depleted after vaccination was modest. We reasoned that the *ura5*Δ*ZNF2*^oe^ strain performed poorly because the strain might have been cleared too rapidly by the host and thus essentially acted similarly to heat-inactivated cells at a dose lower than our standard dose. In contrast, vaccination with the live *sre1*Δ*ZNF2*^oe^ strain showed strong protection in A/Jcr mice with or without CD4^+^ T cell depletion at the time of fungal challenge. No *sre1*Δ*ZNF2*^oe^ cells were recovered from surviving animals after the otherwise lethal challenges in all animals tested, indicating that this strain could be safe to use even in mice with their CD4^+^ T cells depleted. Remarkably, CBA/J and A/Jcr CD4-depleted mice, mimicking AIDS patients, were largely protected against cryptococcosis when vaccinated with the live *sre1*Δ*ZNF2*^oe^ cells. These results are encouraging, as they suggest that vaccination could work effectively not only for hosts who may become immunocompromised before cryptococcal infection occurs (e.g., people living with HIV prior to becoming AIDS patients) but also for hosts with a preexisting immune deficiency (e.g., AIDS patients).

In conclusion, we show evidence of long-term effective protection and relapse prevention by vaccination with HK *ZNF2*^oe^ cells. We also show that vaccination with HK *ZNF2*^oe^ cells does not cause any adverse effects in hosts with persistent nonsymptomatic lung infections. More importantly, we showed that vaccination could work for immunocompetent hosts who become immunocompromised by the time of cryptococcal infection and also for hosts with a preexisting immune deficiency. The findings have important implications for cryptococcal vaccine development, given the predominance of cryptococcosis in AIDS patients and the high risk of this disease in people living with HIV.

Developing effective cryptococcal vaccines remains one of the most important and challenging research areas that could help prevent and manage deadly cryptococcal infections (51). In preclinical studies, multiple vaccines are protective in hosts with a competent immune system; however, few have shown efficacy when the host’s immune system is impaired (52–54). These vaccines have been shown to be protective in various mouse models of cryptococcal meningitis, including animals depleted of CD4^+^ T cells (reviewed in reference 48). Vaccination with recombinant glycosylphosphatidylinositol (GPI)-anchored mannoproteins and chitin deacetylases Cda1, Cda2, and Cda3 together with glucan particles also provide a significant survival advantage to mice against cryptococcosis (37). Developing mRNA vaccines is in the works for the near future. Nonetheless, whole-cell vaccines often provide inexpensive and long-lasting immunity that are critical for vaccine success in the resource-poor settings where this disease hits the hardest. So far, effective whole-cell live attenuated or heat-inactivated vaccines include the hyphal *ZNF2*^oe^ strain, the chitosan-deficient *cda1-3*Δ mutant, the sterylglucosidase *sgl1*Δ mutant, and the ubiquitination E3 ligase *fbp1*Δ mutant (30, 36, 47, 55, 56). It would be extremely valuable for the community to assess these strains systematically and identify the best (and possibly a combination of mutations) to move forward to the next stage of vaccine development to combat this deadly fungal disease.

## MATERIALS AND METHODS

### Ethics statements.

This study was performed according to the guidelines of NIH and the University of Georgia Institutional Animal Care and Use Committee (IACUC). The animal models and procedures used were approved by the IACUC (AUP protocol numbers A2017 08-023 and A2020 06-015).

### Murine model of cryptococcosis. (i) Virulence.

Female A/Jcr mice and CBA/J mice 8 to 10 weeks old were purchased from the Jackson Laboratory (Bar Harbor, ME). Most experiments were carried out in A/Jcr mice unless otherwise indicated. For infection, cryptococcal strains were inoculated in 3 mL of YPD medium with the initial inoculum of approximately 10^6^ cells/mL. Cells were cultured at 30°C with shaking at 220 rpm for 15 h. Cells were washed with sterile saline three times and adjusted to a final concentration of 2 × 10^5^ cells/mL. Mice were sedated with ketamine and xylazine via intraperitoneal injection and then inoculated intranasally with 50 μL fungal cell suspension (1 × 10^4^ cells per animal) as previously described (31, 43, 57, 58). After infection, animals were monitored daily for disease progression, including weight loss, gait changes, labored breathing, or fur ruffling. For fungal burden measurements, animals were euthanized on the designated day postinfection. For the survival experiments, mice were euthanized when they reached the clinical endpoint. All the surviving animals were terminated at the predetermined days for the ending of the experiments.

### (ii) Vaccination.

To prepare fungal cells used for vaccination, each strain was inoculated in 3 mL of YPD medium at 10^6^ cells/mL. Cells were cultured at 30°C with shaking at 220 rpm for 15 h. The fungal cells were washed with sterile saline three times and adjusted with saline to a final concentration of cell suspension of 1 × 10^8^ cells/mL (live vaccination with 5 × 10^6^ cells per animal), 2 × 10^8^ cell/mL (HK vaccination at the typical dose of 1 × 10^7^ cells per animal), or 1 × 10^9^ cell/mL (HK vaccination at the high dose of 5 × 10^7^ cells per animal). For inactivation of cells for vaccination, the cell suspension was heated at 95°C for 20 to 25 min. Mice were sedated with ketamine and xylazine via intraperitoneal injection and then inoculated intranasally with 50 μL cell suspension using previously described procedures (30). Three vaccination regimens were used in this study. (i) For live-cell vaccination, mice were vaccinated twice with a live strain, at day −37 and day −12. (ii) For vaccination with heat-inactivated cells, mice were vaccinated with heat-killed cells twice, at day −32 and at day −7 (30). (iii) In another regimen of vaccination with heat-inactivated cells, mice were vaccinated with heat-killed cells twice, at day −37 and at day −12 (30). For infection, live H99 or BC1076 cells with the initial inoculum of 10^6^ cells/mL were cultured in 3 mL of YPD medium at 30°C with shaking at 220 rpm for 15 h. Cells were washed with sterile saline 3 times and adjusted to a final concentration of 2 × 10^5^ cell/mL (1 × 10^4^ cells/animal). The infection process was the same as previously described.

### (iii) T cell depletion.

Mice were depleted of CD4^+^ and/or CD8^+^ T cell subsets via intraperitoneal administration of anti-CD4 (GK1.5, rat IgG2b) and anti-CD8a (2.43, rat IgG2b) antibodies (Bio X Cell, New Hampshire) (59). Each mouse received 200 μg per 20 g body weight of GK1.5 and/or 2.43 of control rat IgG2b antibodies in a volume of 200 μL phosphate-buffered saline (PBS) 48 h prior to infection or prior to vaccination depending on the regimen used (see the figures and their legends for details). These mice also received these antibodies weekly thereafter during the observation period. The efficiency of T cell depletion was monitored by flow cytometry on peripheral blood samples. Briefly, red blood cells (RBC) were lysed by treatment with Ammonium-Chloride-Potassium (ACK) lysing solution, and cells were washed with PBS. Cells were stained with a mixture of anti-mouse immunoglobulin antibodies, including Fc block (unlabeled anti-CD16/32; clone 93; Invitrogen), anti-T cell receptor β (TCRβ) (fluorescein isothiocyanate [FITC]; clone H57-597; eBioscience), anti-CD4 (allophycocyanin [APC]; clone RM4-5; eBioscience), and anti-CD8α (efluor 450; clone 53-6.7; eBioscience) for 10 min at 4°C in the dark. Cells were washed with cold PBS and immediately analyzed on a Novocyte Quanteon flow cytometer. The percentage of TCRβ^+^ T cells expressing either CD4 or CD8 was calculated.

### (iv) Fungal burden analysis.

At the indicated time of euthanasia or at the termination of the survival experiments (DPI 60), the lungs, kidneys, and brains of the euthanized mice were dissected. The dissected organs were homogenized in 2 mL cold PBS buffer using an IKA-T18 homogenizer with the setting for each type of organ that we described previously (30, 57). The tissue suspensions were serially diluted (10×), plated on YNB solid medium, and incubated at 30°C for 2 days, so that the colonies became visible for counting CFU.

### Strains and culture conditions.

All strains were stocked in 15% glycerol and stored at −80^0^C. Fresh cultures were used for experiments. All strains used in this study were serotype A isolates in the H99 background. Fungal strains were maintained on YPD at 30°C unless indicated otherwise.

### Generation of mutants.

The *ura5*Δ and *sre1*Δ mutant strains were generated in the LW10 background where *ZNF2* expression is driven by the *GPD1* promoter (28). To create these strains, we deleted the *URA5* or the *SRE1* open reading frame (ORF) from the *ZNF2*^oe^ strain using the CRISPR-Cas9 transient expression system TRACE (60). Transformants were validated using stability testing, diagnostic PCR screening for the absence of the *URA5* or *SRE1* open reading frame. The overexpression of *ZNF2* in these strains was confirmed by the observation of filamentation in YPD medium. Primers used in this study are listed in Table S1.

### Phenotypic assays of the mutant strains.

The indicated strains were cultured in liquid YPD medium at 30°C with shaking at 220 rpm for 12 h. Cells were washed with double-distilled water (ddH_2_O), adjusted to the same cell density (optical density at 600 nm [OD_600_] = 1.0) with a spectrophotometer, and then serially diluted. For phenotypic analyses, 3 μL of serial dilutions was spotted onto various agar media. To test thermotolerance, cells of the indicated strains were spotted onto YPD medium and incubated at 22°C, 30°C, or 37°C for 2 days. YPD medium supplemented with Congo red (0.05%) was used to test the susceptibility of cells to cell wall stress. To test CO_2_ tolerance, cells were incubated on YPD medium in the ambient air or in 5% CO_2_ at 30°C for 2 days. To test auxotrophy of the *ura5*Δ mutants, cells were grown on minimal YNB medium, RPMI medium, or LIM at 30°C for 2 days. To test the ability of cells to grow under hypoxic conditions, cells were grown on yeast extract sucrose (YES) medium supplemented with 0.6 mM CoCl_2_ (hypoxia-mimicking condition) at 30°C for 2 days.

### Immunofluorescence staining.

Cryptococcal cells were cultured in liquid YPD medium at 30°C with shaking at 220 rpm overnight. The cells were washed twice with sterile ddH_2_O, suspended in PBS, and enumerated using a hemocytometer to achieve a final cell density of 2 × 10^7^ cells/mL. A 1-mL aliquot of cells was fixed with 4% formaldehyde for 5 min at room temperature. Then, fixed cells were washed with PBS and blocked in 1 mL of 1% bovine serum albumin (BSA) in PBS at room temperature for 1 h. After blocking, cells were washed twice with PBS and resuspended in 100 μL of a 1:10 dilution of HK *ZNF2*^oe^ cell serum with 1% BSA. The serum was collected from HK *ZNF2*^oe^ cell-vaccinated mice as previously described (31). Cells were incubated at room temperature for 1 h and then washed twice with PBS. Cells were then incubated with100 μL of goat IgG/IgM anti-mouse secondary antibody conjugated to Cy3 (1:200 dilution) for 1 h at room temperature. Following that, cells were washed twice and resuspended in PBS before imaging.

### Microscopy.

Regular light microscopy was performed using the Olympus CX41 microscope (Olympus Life Sciences, Tokyo, Japan). Colony images were photographed with a SZX16 stereoscope (Olympus Life Sciences, Tokyo, Japan). Images were acquired with an AxioCam MRm camera and processed with the software Zen pro.

Fluorescent microscopy was performed using a Zeiss Imager M2 microscope (Zeiss, Oberkochen, Germany). Images were acquired with an AxioCam MRm camera and processed with the software Zen pro. The fluorescence intensity of 50 individual cells from each strain imaged at ×63 magnification was quantified using Zen 2.6 Blue edition software (Zeiss, Oberkochen, Germany). Fluorescence intensity was quantified using the Zen Histo definition quantification software application. Each cell and its background were selected using the circular selection tool, and the average fluorescence intensity within that circle was recorded. The fluorescence intensity of the background around each cell was measured and served as a blank. The fluorescence intensity of each cell was normalized by subtracting the fluorescence intensity of the cell’s associated blank.

### Statistical analysis.

Statistical significance of the survival data between different groups was assessed by the Gehan-Breslow-Wilcoxon test. One-way analysis of variance (ANOVA) was used in the fungal burden studies. All statistical analyses were performed using GraphPad Prism version 8.11, with *P* values lower than 0.05 being considered statistically significant.

### Data availability.

All of the data supporting this study are presented herein, and the reported fungal strains generated for this study are available on request.
