# Supplementary figures and images for: Vaccination with a ZNF2oe Strain of Cryptococcus Provides Long-Lasting Protection against Cryptococcosis and Is Effective in Immunocompromised Hosts
Source: Infect Immun. 2023 Jun 20;91(7):e00198-23. doi: 10.1128/iai.00198-23 (PMC10353382; doi:10.1128/iai.00198-23)

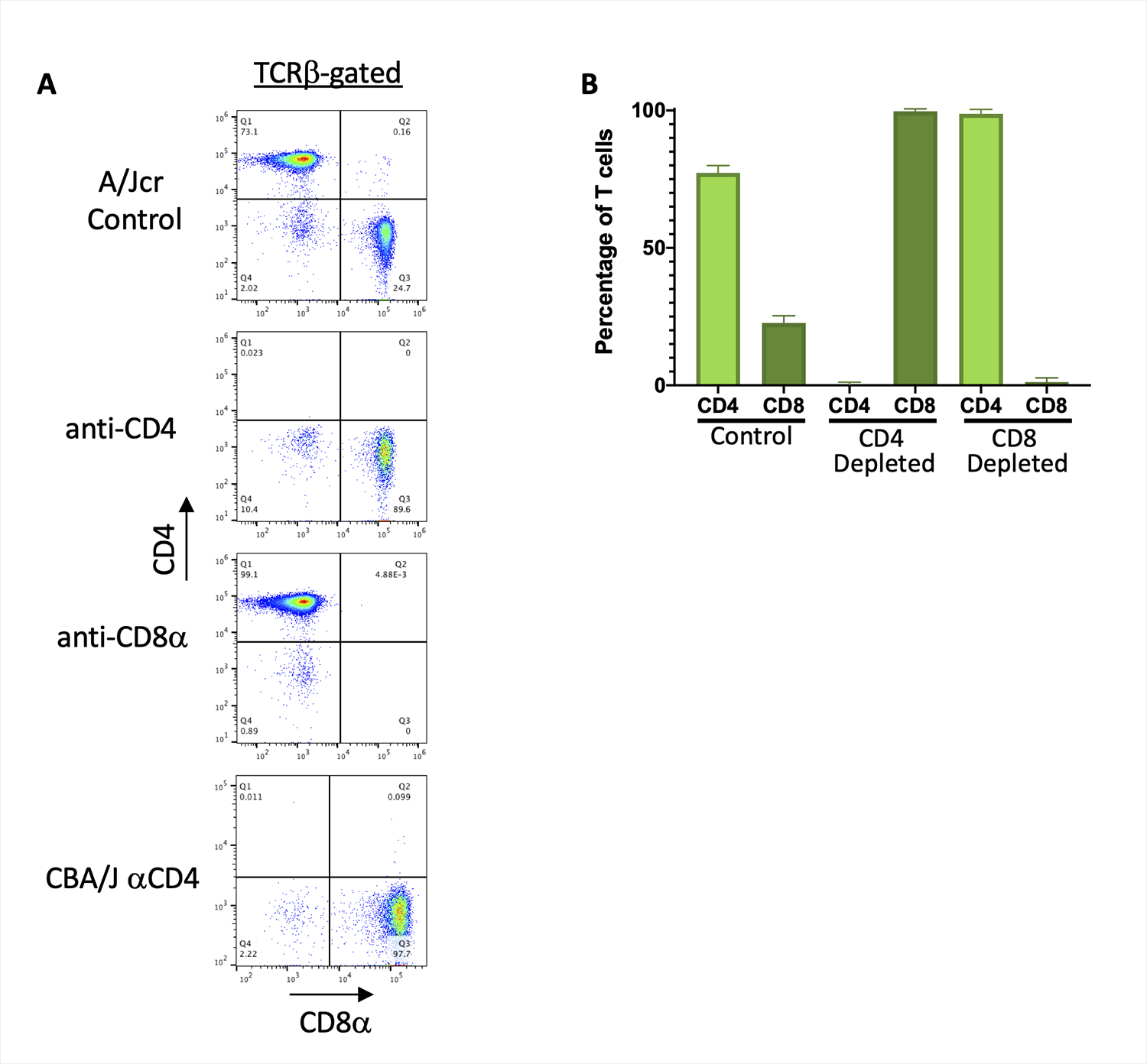

Supplement: Supplemental file 1 — Fig. S1. Download iai.00198-23-s0001.tif, TIF file, 8.0 MB [file iai.00198-23-s0001.tif]

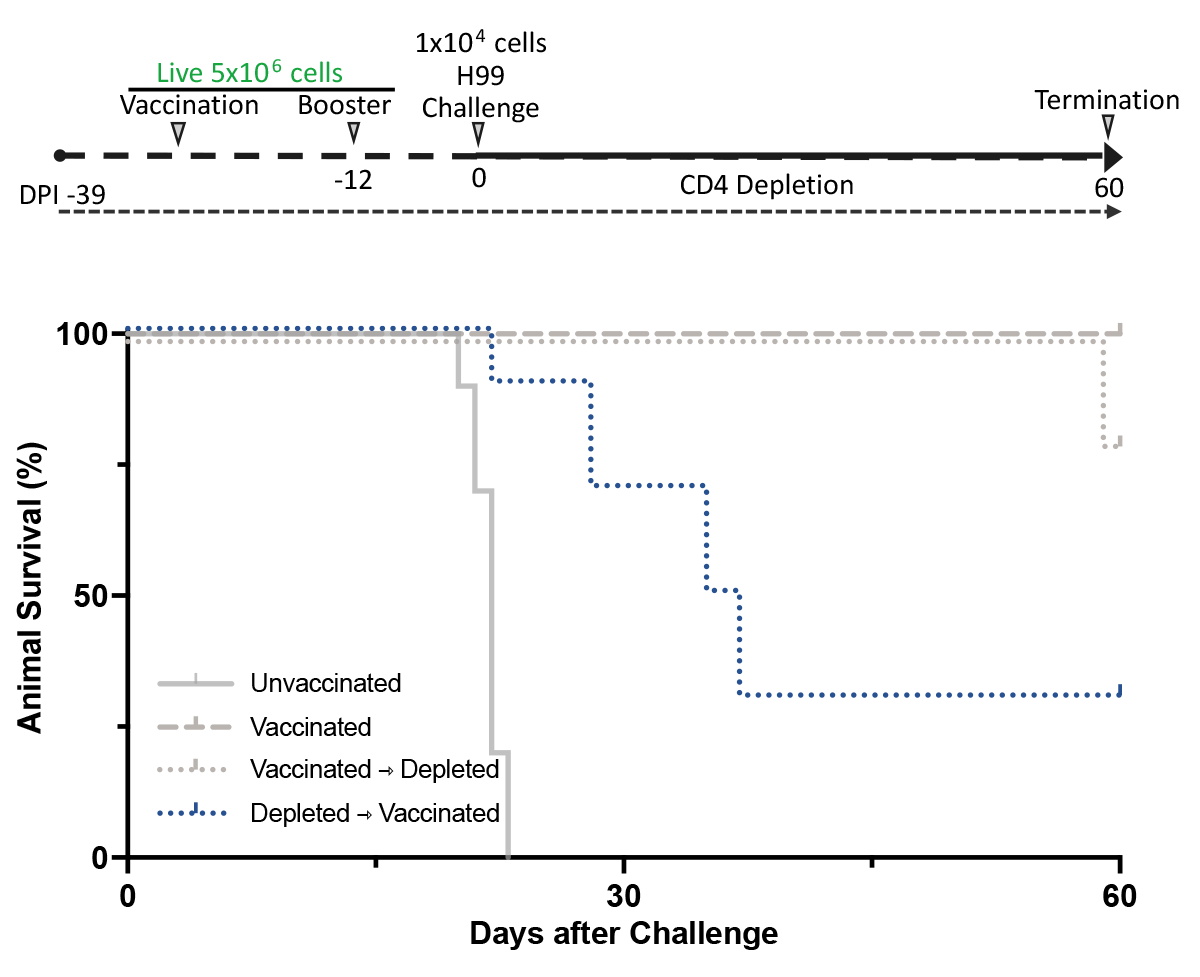

Supplement: Supplemental file 2 — Fig. S2. Download iai.00198-23-s0002.tif, TIF file, 0.3 MB [file iai.00198-23-s0002.tif]
