# Supplementary material for: Vaccination with a ZNF2oe Strain of Cryptococcus Provides Long-Lasting Protection against Cryptococcosis and Is Effective in Immunocompromised Hosts
Source: Infect Immun. 2023 Jun 20;91(7):e00198-23. doi: 10.1128/iai.00198-23 (PMC10353382; doi:10.1128/iai.00198-23)
Supplement: Supplemental file 3 — Legends of Fig. S1 and S2. Download iai.00198-23-s0003.pdf, PDF file, 0.1 MB [file iai.00198-23-s0003.pdf]

Supplemental Figure 1: **CD4 and CD8 T cells are effectively being depleted by the antibody GK1.5 and 2.43 respectively.** The efficiency of T cell depletion in mice was monitored by flow cytometry on peripheral blood samples. **A**. Representative images of the staining for $CD4^+$ and $CD8^+$ T cells of A/Jcr mice and $CD4^+$ T cells of CBA/J mice within the $TCRb^+$ gate. **B**. Calculated percentage of CD4 and CD8 T cells in A/Jcr control mice or mice depleted of $CD4^+$ or $CD8^+$ T cells.

Supplemental Figure 2: **CD4-deficient A/Jcr mice vaccinated with live *sre1ΔZNF2*^{oe} cells have minor protection.** 10 A/Jcr mice were depleted of their $CD4^+$ T-cells then vaccinated with live *sre1*D*ZNF2*^{oe} cells. Mice depleted with $CD4^+$ T-cells then vaccinated, shows minor protection against wild type H99. Gray = Data from main figure. Control: Figure 1B Vaccinated: Figure 4D
